# Study on the efficacy and safety of neoadjuvant immunotherapy combined with chemotherapy regimen for III–IVA esophageal squamous cell carcinoma post-surgery

**DOI:** 10.1186/s13019-024-02500-9

**Published:** 2024-01-25

**Authors:** Chunyang Li, Pengyi Yu, Hong Li, Xin Yang, Jun Wang, Bo Jiang

**Affiliations:** 1 Department of Thoracic Surgery, The First People’s Hospital of Chang-Zhou City, The Third Affiliated Hospital of Soochow University, 185 Juqian Road, Jiangsu, 213003 China; 2 Department of Oncology, The First People’s Hospital of Chang-Zhou City, The Third Affiliated Hospital of Soochow University, 185 Juqian Road, Jiangsu, 213003 China

**Keywords:** Neoadjuvant therapy immunotherapy, Esophageal squamous cell carcinoma, Safety

## Abstract

**Background and aims:**

The treatment of esophageal squamous cell carcinoma is still controversial, and neoadjuvant chemotherapy combined with immunotherapy is a hot topic of current research. We investigated the recent efficacy and surgical safety of patients with III–IVA esophageal squamous cell carcinoma after neoadjuvant regimen of paclitaxel + cisplatin/nedaplatin/carboplatin + sindilizumab, to provide a theoretical basis for evaluating the feasibility of surgery after neoadjuvant therapy.

**Methods:**

The clinical data of patients with stage III–IVA esophageal squamous cell carcinoma admitted from January 2022 to April 2023 at our hospital were collected for retrospective analysis. The patients were divided into the neoadjuvant combination surgery group (34 patients with the regimen of paclitaxel + cisplatin/nedaplatin/carboplatin + sintilimab two/three cycles of preoperative neoadjuvant therapy) and surgery-only group (36 patients). Statistical analysis was performed to compare the differences between both groups particularly for intraoperative bleeding, operative time, incidence of postoperative pulmonary complications, laryngeal recurrent nerve injury, thoracic duct injury, anastomotic fistula, and postoperative hospital days. Additionally, the pCR/MPR rates of the neoadjuvant group were analysed.

**Results:**

Significant differences were present in the clinical and pathological staging before and after neoadjuvant treatment (*P* ≤ 0.001). The neoadjuvant group had a pCR rate of 26.47% and an ORR rate of 88.23%. No significant differences were discovered in R0 resection rate between both groups, as well as intraoperative bleeding, operative time, intraoperative laryngeal recurrent nerve injury rate, thoracic duct injury rate, postoperative anastomosis incidence, postoperative hospital days, and postoperative lung infection incidence (*P* > 0.05).

**Conclusions:**

The neoadjuvant immune combination chemotherapy regimen had considerable tumor regression and pathological remission benefits, without reducing the safety of surgery, possibly presenting as a new treatment plan.

## Introduction

### Esophageal cancer incidence and neoadjuvant therapy

Esophageal cancer (EC) is one of the most common malignancies in the world, especially in East Asia, with China accounting for about half of the global incidence and mortality [1]. Esophageal squamous cell carcinoma (ESCC) accounts for more than 90% of esophageal cancers in China. Over the past 40 years, great achievements have been made in the treatment of ESCC. However, when patients with III–IVA ESCC were treated according to standard treatment guidelines, the overall survival is unsatisfactory, warranting further exploration of better treatment options.

Compared with postoperative adjuvant therapy, preoperative neoadjuvant therapy has multiple advantages, including a high completion rate, preoperative tumor downstaging, killing micrometastases, improving surgical resection rate and complete pathological remission rate, and more [2]. Neoadjuvant radiotherapy (nRT), neoadjuvant chemotherapy (nCT), neoadjuvant chemoradiotherapy (nCRT), and neoadjuvant immunotherapy are currently used in clinical practice. In combined chemotherapy/radiotherapy regimens, the neoadjuvant regimen commonly consists of two cycles, operated at 6–8 weeks postoperatively, with varying survival rates, complication rates, and postoperative mortality.

### Immunotherapy and neoadjuvant immune combination chemotherapy for esophageal cancer

Immunotherapy has developed rapidly in recent years, and on July 30, 2019, Pembrolizumab was officially approved for patients with relapsed, stage III–IVA, or metastatic ESCC treated with first-line or multiline systemic therapy, and positive for PD-L1 expression. Many studies have demonstrated the effective role of immunotherapy in the treatment of stage II oesophageal cancer, and some investigators have also combined immunotherapy with neoadjuvant therapy. These clinical trials have contributed to the development of neoadjuvant therapy for ESCC and facilitated the further search for the best standard treatment modality.

Nevertheless, neoadjuvant therapy for stage III–IVA ESCC is currently the subject of multiple controversies. A series of studies have demonstrated that neoadjuvant therapy can improve R0 resection and pCR rates, prolong patient OS, and improve prognosis, but may also increase the surgical difficulty and operative risks. Some studies have shown that neoadjuvant therapy may improve OS but increase the risk of postoperative death in patients. In addition, the choice of neoadjuvant regimens, immune drugs, chemotherapy regimens, radiation doses, intervals between neoadjuvant and surgery, the extent of surgical lesion, and lymph node clearance need further study [3].

## Research objective and methodology

### Study subjects

Patients admitted to our hospital with stage III–IVA esophageal squamous cell carcinoma who met the inclusion and exclusion criteria between January 2022 and April 2023 were included for retrospective analysis. According to their treatment regimen, 34 patients who underwent radical esophageal cancer surgery after two/three cycles of the treatment regimen of paclitaxel + cisplatin/nedaplatin/carboplatin + sintilimab represented the observation group, and 36 patients who underwent radical esophageal cancer surgery alone represented the control group.

In the observation group, there were 33 males and 1 female at an average age of 64.56 ± 6.805 years, five cases of upper segment esophageal cancer, 20 cases of middle segment esophageal cancer, and nine cases of lower segment esophageal cancer. Of these patients, 27 had clinical stage IIIB cancer and seven patients’ cancers were at stage IVA. The control group consisted of 30 males and 6 females, at an average age of 67.64 ± 7.337 years. Among them, one had upper segment esophageal cancer, 25 had middle segment esophageal cancer, 10 had lower segment esophageal cancer, while five and 31 patients had stage IIIA and IIIB cancer, respectively.

### Inclusion and exclusion criteria

#### Inclusion criteria

1. Aged 18–85 years (including 18 and 85 years);
2. Patients with clinical stage III–IVA esophageal cancer as assessed by ultrasound endoscopy, computerized tomography (CT)/magnetic resonance imaging (MRI), and other imaging;
3. Had undergone two/three cycles of neoadjuvant treatment with paclitaxel + cisplatin/nedaplatin/carboplatin + sintilizumab;
4. Have successfully undergone surgery after neoadjuvant therapy;
5. The surgeries were all done in the same treatment group;

Note:

1. Conditions (3) and (4) only apply to the neoadjuvant group.
2. Staging is based on the TNM staging criteria for esophageal cancer, 8th edition (AJCC, 2017 edition).

#### Exclusion criteria

1. Previous or concurrent other malignancies;
2. A history of:
  - immunodeficiency disease, or
  - organ transplantation;
3. Patients with postoperative pathological staging confirmed as stage IVB;
4. Incomplete case information.

### Treatment method

#### Preoperative neoadjuvant therapy

In the observation group, the neoadjuvant immune combination chemotherapy regimen was paclitaxel(200 mg) + cisplatin(100 mg/m^2^)/nedaplatin(100 mg/m^2^)/carboplatin(400 mg/m^2^) + sintilimab(200 mg) in one or two days. One cycle is 21 days, and patients were treated symptomatically in case of adverse reactions.

#### Surgical treatment

In the control group, oesophagectomy was performed electively after the exclusion of contraindications. In the observation group, oesophagectomy was performed electively after 4–6 weeks of systemic assessment, after two/three cycles of neoadjuvant therapy. The Ivor-Lewis approach or McKeown approah was chosen according to the patient's specific situation. Two-field lymph node dissection or three-field lymph node dissection were performed as standard. Both groups were operated by the same treatment group.

### Efficacy assessment and observation index

1. Neoadjuvant efficacy was evaluated based on the International Union Against Cancer efficacy criteria and classified according to relevant imaging criteria:
  - Complete remission (CR);
  - Partial remission (PR);
  - Stable disease (SD);
  - Disease progression (PD).
    The tumor downgrading criteria were in accordance with the TNM staging criteria for esophageal cancer, 8th edition (AJCC, 2017 edition).
2. The relevant indexes to evaluate the recent efficacy of patients were mainly pCR/MPR rate.
3. The relevant indexes used to evaluate the safety of patients' surgery consisted mainly of intraoperative bleeding, operation time, postoperative pulmonary complication rate, laryngeal recurrent nerve injury, thoracic duct injury, anastomotic fistula, and postoperative hospitalization days.

### Statistical methods

Statistical analyses were conducted using the SPPS 25.0 software, and the measures were tested for normal distribution. For normally distributed data, analysis was expressed as mean plus standard deviation (−x ± s), and data between two groups were expressed using independent samples t-test. For skewed data, analysis was expressed as median plus quartiles (P25, P75), and the data between two groups were expressed using the Mann–Whitney U test. Measures were expressed as percentages, and comparisons were made using the Chi-Square test. The test level *α* = 0.05 was set, and *P* < 0.05 was regarded as statistically significant.

## Results

### Comparisons of general information

Statistical analysis of the neoadjuvant group and the surgery-only group in terms of gender, age, tumor location, clinical stage, pathological type, surgical approach, lymph node dissecton, and anastomosis, respectively. Statistical differences were not present between both groups in any of the above factors (*P* > 0.05). For details, see Table 1.

**Table 1:** Comparison of general information

		Observation group	Control group	*P*-value
Gender	Male	33 (97.06%)	30 (83.33%)	0.107
	Female	1 (2.94%)	6 (16.67%)	
Age		64.56 ± 6.805	67.64 ± 7.337	0.073
Tumor location	Upper thoracic	5 (14.71%)	1 (2.78%)	0.239
	Middle thoracic	20 (58.82%)	25 (69.44%)	
	Lower thoracic	9 (26.47%)	10 (27.78%)	
Clinical stage	IIIA	0	5 (13.89%)	0.239
	IIIB	27 (79.41%)	31 (86.11%)	
	IVA	7 (20.59%)	0	
Pathology type	Ulceratice	23 (67.65%)	23 (63.89%)	0.725
	Mushroom	5 (14.71%)	3 (8.33%)	
	Constrictive	5 (14.71%)	9 (25.00%)	
	Medullary	1 (2.94%)	1 (2.78%)	
Surgical approach	Ivor-Lewis	9 (26.47%)	10 (27.78%)	0.210
	McKeown	25 (73.53%)	26 (72.22%)	
Lymph node dissection	Two-field	9 (26.47%)	10 (27.78%)	0.210
	Three-field	25 (73.53%)	26 (72.22%)	
Anastomosis	Cervical	25 (73.53%)	26 (72.22%)	0.210
	Thoracic	9 (26.47%)	10 (27.78%)	

### Comparison of other information between both groups

Statistical analysis of the R0 resection rate, intraoperative bleeding, operative time, intraoperative laryngeal recurrent nerve injury rate, thoracic duct injury rate, postoperative anastomosis incidence, postoperative hospital days, and postoperative pulmonary infection incidence. No statistical differences were present between both groups in any of these factors (*P* > 0.05). Further details are shown in Table 2.

**Table 2:** Comparison of other information between both groups

	Observation group	Control group	*P*-value
R0 resection rate	33/34 (97.06%)	34/36 (94.44%)	1.0
Intraoperative bleeding volume (ml)	100 (92.5,150)	100 (100,200)	0.370
Surgery time (min)	212 (192, 240.72)	205 (188.25,241)	0.540
Recurrent laryngeal nerve injury rate	1/34 (2.94%)	1/36 (2.78%)	1.0
Thoracic duct injury rate	0/34	0/36	1.0
Anastomotic fistula rate	3/34 (8.82%)	6/36 (16.67%)	0.479
Number of postoperative hospital days (d)	12 (9.25,16)	14 (10,20.75)	0.289
Postoperative lung infection rate	7/34 (20.59%)	7/36 (19.44%)	0.905

We found postoperative anastomotic fistulae as well as pulmonary infections in both groups, but none were life-threatening. Five patients developed anastomotic fistula of Clavien-Dindo grade III, which improved after surgical management. There were no significant differences in complications between the two groups (Table 3, 4).

**Table 3:** Details of surgical complications and the number of cases between both groups

Complications and Clavien-Dindo classification	Observation group	Control group	*P*-value
Anastomotic fistula	3	6	1.0
Grade II	1	3	
Grade III	2	3	
Postoperative lung infection rate	7	7	1.0
Grade II	7	7	
Grade III	0	0

**Table 4:** Information related to the neoadjuvant treatment group before and after neoadjuvant treatment

		Pre-neoadjuvant therapy	Post-neoadjuvant therapy	*P*-value
T stage	0	0	8 (21.53%)	0.001
	1	0	4 (11.76%)	
	2	1 (2.94%)	7 (20.59%)	
	3	34 (97.06%)	15 (44.12%)	
	4	0	0	
N stage	0	0	19 (55.58%)	< 0.001
	1	3 (8.82%)	6 (17.65%)	
	2	24 (70.59%)	6 (17.65%)	
	3	7 (20.59%)	3 (8.82%)	
Clinical stage	1	0	13 (38.24%)	< 0.001
	2	0	6 (17.65%)	
	3	27 (79.41%)	12 (35.29%)	
	4	7 (20.59%)	3 (8.82%)	
pCR			9 (26.47%)	
MPR			4 (11.76%)	
CR			9 (26.47%)	
PR			21 (61.76%)	
SD			4 (11.76%)	
PD			0	
TRG (tumor regression grade)	0		9 (26.47%)	0.019
	1		2 (5.88%)	
	2		8 (21.53%)	
	3		15 (44.12%)	

### Information related to the neoadjuvant treatment group before and after neoadjuvant treatment

**Fig. 1:**
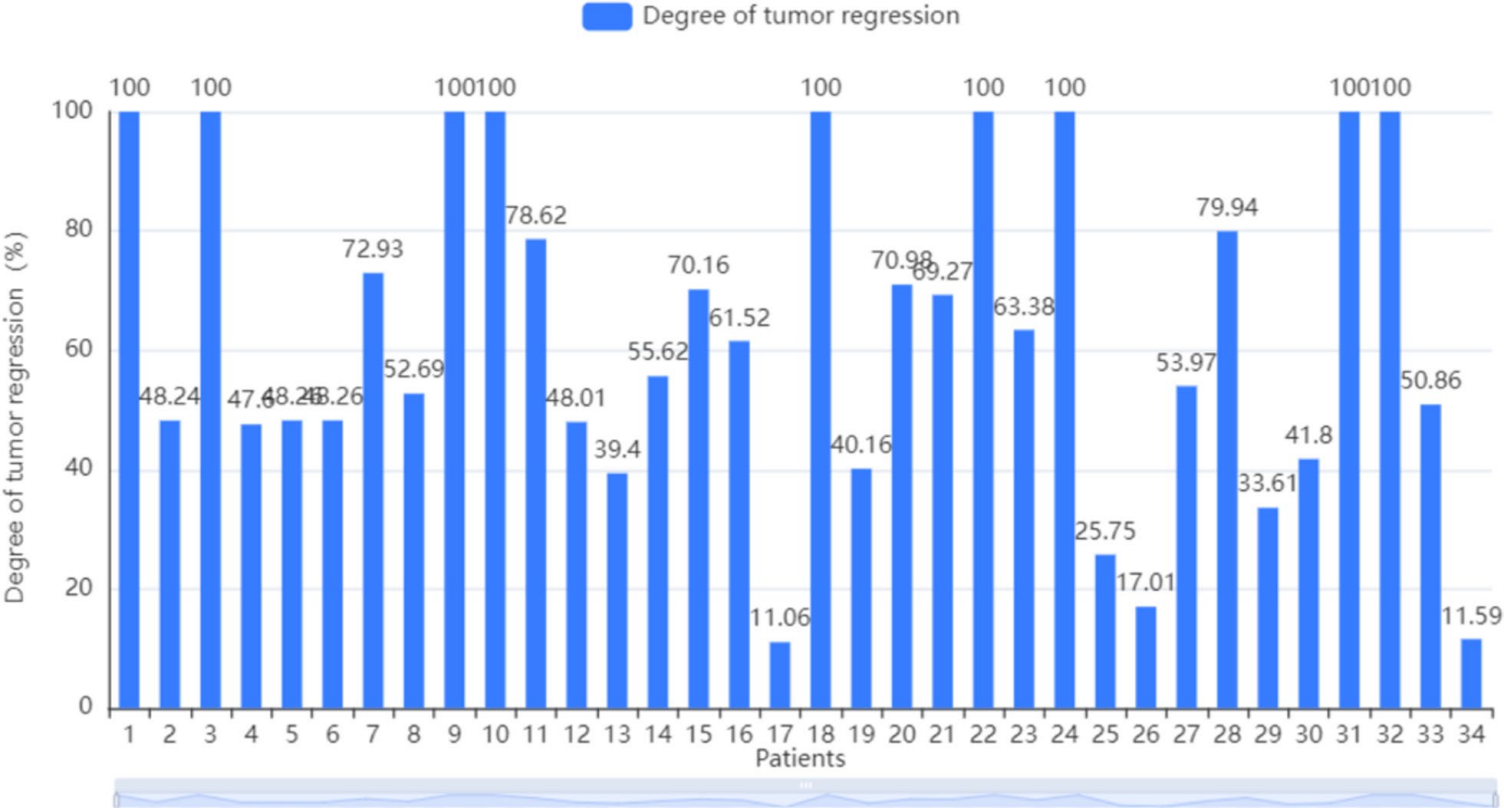
Degree of tumor regression

Table 5 showed that the neoadjuvant therapy group did not develop immune-related pneumonia, myocarditis, and liver and kidney damage. Some patients had minor endocrine disorders,dermatitis, and leukopenia, which were controlled with further medication and did not affect subsequent treatment (Fig. 1).

**Table 5:** Adverse events of the neoadjuvant therapy group

Adverse events	Rate
Pneumonia	0/34
Myocarditis	0/34
Liver damage	0/34
Renal damage	0/34
Endocrine disorder	3/34
Dermatitis	1/34
Leucopenia	6/34

Analysis of the TNM staging of the neoadjuvant group before and after receiving neoadjuvant therapy, revealed significant differences in pathological and clinical staging before and after neoadjuvant therapy (*p* ≤ 0.001). Statistical calculations yielded a pCR rate of 26.47%, an MPR rate of 11.76%, 9 patients had achieved CR, 21 patients achieved PR, four patients had SD, and the ORR was 88.23% for the neoadjuvant group. Pathological results suggested that nine patients achieved a TRG grade of 0, who patients had TRG grade of 1, eight patients had a TRG grade of 2, and the remaining 15 patients only achieved a TRG grade of 3. See Table 4 for details.

### Comparison of different pathological patterns in the neoadjuvant group

Analysis of tumour regression in the neoadjuvant group based on different pathological forms, found that ulcerated lesions had the highest pCR rate and a TRG grade of 0. See Table 6 for more details.

**Table 6:** Comparison of different pathological patterns in the neoadjuvant group

	Ulceratice	Mushroom	Constrictive	Medullary
Numbers	23	5	5	1
pCR	8 (34.78%)	0	1 (20.00%)	0
MPR	2 (8.70%)	2 (40.00%)	0	0
CR	8 (34.78%)	0 (	1 (20.00%)	0
PR	11 (47.83%)	5 (100.00%)	4 (80.00%)	1 (100.00%)
SD	4 (11.39%)	0	0	0
PD	0	0	0	0
TRG0	8 (34.78%)	0	1 (20.00%)	0

## Conclusion

Immunotherapy has become an emerging oncology treatment modality, with intensive research in the field of EC in recent years. Numerous studies have used immunotherapy as a post-operative adjuvant treatment and pre-operative neoadjuvant treatment of EC [4–8].

In the KEYNOTE-180 trial, Pembrolizumab was demonstrated to be a second-line treatment for advanced esophageal cancer patients with a high expression of PD-L1 [9]. In the KEYNOTE-181 study, Pembrolizumab was found to be superior to chemotherapy in patients with advanced esophageal cancer, with PD-L1 ≥ 10 detected by Combined Positive Score (CPS) [10]. The subgroup analysis revealed that Pembrolizumab showed better efficacy and fewer adverse events in Asian ESCC patients [11]. In the ESCORT study, Camerelizumab showed similar effect, which represents a alternative to standard second-line treatment for Chinese ESCC patients [12]. In the ATTRACTION-3 study, Nivolumab significantly prolonged overall survival and had a favorable safety profile compared to chemotherapy [13]. And in the ATTRACTION-4 study, Nivolumab in combination with oxaliplatin as a treatment regimen significantly improved progression-free survival. However, overall survival was not improved [14].

The effective role of PD-1 inhibitor in the treatment of advanced oesophageal cancer inspired some investigators to combine immunotherapy with neoadjuvant therapy in resectable ESCC. Yamamoto et al. conducted a study to investigate the efficacy and safety of preoperative Nivolumab in combination with chemotherapy in III–IVA EC [15]. In China, the NICE study showed the addition of Camrelizumab to the neoadjuvant chemotherapy treatment was well tolerated and it was noticed that pCR was independent of PD-L1 levels [16]. Wang et al. further demonstrated a satisfactory response to neoadjuvant immunotherapy combined with chemotherapy regimens in Chinese ESCC patients [17]. Lin et al. retrospectively analyzed the safety and efficacy of Pembrolizumab for neoadjuvant treatment of ESCC and confirmed the therapy’s ability to produce higher ORR(Objective Response Rate), MPR(Major Pathologic Response), pCR, and R0 resection rate [18]. Shang et al. conducted a clinical trial to evaluate the safety and efficacy of Pembrolizumab in combination with paclitaxel cisplatin as a neoadjuvant treatment option for stage III esophageal cancer [19]. Once data from these clinical trials are available, they will facilitate the development of neoadjuvant treatments for ESCC and facilitate further exploration of the best standard treatment modalities.

These studies have confirmed the therapeutic benefits of immunotherapy in the field of oesophageal cancer, but further refinement and more data are needed.

Salas-Benito et al. suggest that immunotherapy and chemotherapy may work synergistically to promote immunogenic tumor cell death, is anti-angiogenesis, and causes selective depletion of myeloid immunosuppressive cells and lymphocytopenia. These effects reduce regulatory T cells that free up space for the proliferation of effector T cells. However, current chemotherapy regimens are not optimized for this mechanism [20]. Outcomes from studies involving immune checkpoint inhibitors and clinical trials led to the development of the concept of neoadjuvant immunotherapy, and a more in-depth study of the molecular biology of EC, to further improve the prognosis of patients [21].

In addition, neoadjuvant immunotherapy combined with chemotherapy as a regimen can be damaging to tissues. The combination therapy may lead to adhesions and edema of lesions with surrounding tissues, increasing the risk and difficulty of surgical resection. It may also increase the likelihood of damage to associated anatomical structures and other postoperative complications during the intra-operative freeing of the thoracic duct and recurrent laryngeal nerve [22]. Prolonged chemotherapy and immunotherapy may also deteriorate the patient's general condition, leading to an increased likelihood of postoperative complications or surgery intolerance.

However, based on current data, statistical significance was absent in the safety profile of surgery between patients treated with neoadjuvant and surgery combination and surgery alone, which may be related to the number of subjects included. Furthermore, although the overall benefit of neoadjuvant chemotherapy combined with immunotherapy is substantial, a small proportion of patients who are insensitive to chemotherapy and immune drugs were unable to achieve good tumor regression and pathological remission. In these patients, receiving 2–3 cycles of neoadjuvant therapy may instead result in missing out on the optimal time for surgery or experiencing further tumor progression, thus negatively affecting their prognosis.

Zhang [23], Lv [24], Duan [25], and others have investigated the survival benefit of sintilimab in neoadjuvant chemoimmunotherapy for EC, where relatively high pCR rates and safety profiles were reported, but further study is still needed. The current study included 34 patients who met the criteria for post-neoadjuvant surgery, while 36 patients underwent surgery alone. The data from both groups were analyzed by excluding differences in gender, age, location of the lesion, and pathological stage. The R0 resection rate and intraoperative bleeding, operative time, intraoperative laryngeal recurrent nerve injury rate, thoracic duct injury rate, postoperative anastomosis incidence, postoperative hospital days, and postoperative lung infection incidence were statistically analyzed, and no differences were revealed for any of these factors (*P* > 0.05).

It is deduced that neoadjuvant immunotherapy in combination with chemotherapy did not lead to increased risks associated with surgery and postoperative complications, which may provide a theoretical basis for the safety of neoadjuvant chemotherapy in combination with immunotherapy. Further analysis of the outcomes from the neoadjuvant group after neoadjuvant treatment revealed a significant difference in pathological and clinical staging before and after neoadjuvant therapy (*p* ≤ 0.001). Moreover, the neoadjuvant group had a pCR rate of 26.47 and an MPR rate of 11.76, with 26.47% of patients achieving a TRG grade of 0. Significant tumor regression and pathological remission after neoadjuvant therapy were demonstrated by the subject, which coincides with the TD-NICE study [26], and therefore, provides a lead in the development of subsequent treatment plans. However, four patients achieved SD and only 15 patients achieved a TRG grade of 3. Some patients may not be highly sensitive to neoadjuvant chemotherapy combined with immunotherapy, thus, further research is needed to best select patients who will benefit from neoadjuvant chemotherapy combined with immunotherapy [27].

In addition, lung infection was found to be the most frequent postoperative complication in both the neo-adjuvant and the surgery-alone groups, with probabilities of 20.59% and 19.44%, respectively, but no subjects reported severe pneumonia or needed mechanical-assisted ventilation.

Surgery as a treatment for EC is a highly invasive procedure that involves many anatomical structures, especially intra-thoracic surgery, which increases the likelihood of post-operative lung infection. Surgical trauma from receiving neoadjuvant chemotherapy and immunotherapy may induce a series of immune responses, leading to the development of postoperative immune-related complications, especially immune pneumonitis, and immune myocarditis, which are aggressive and difficult to manage. As a result of these complications, monitoring the immune function during the perioperative period, assessing the risk of immune complications, identifying hidden high-risk factors, and minimally invasive surgery are crucial to reducing further damage to the overburdened bodily systems. If symptoms of immune pneumonitis or myocarditis develop postoperatively, they should be managed as early as possible.

There are certain limitations to highlight in this study. Firstly, information about the prognosis of the patients was not considered at the time the current data was reported. Prognosis data is planned to be analyzed upon completion of data collection. Secondly, the study has a small number of subjects, which is due to the single-center nature of the study, with data collection spanning nearly over a year, thus, possibly biased.

However, with available data, ESCC patients treated with neoadjuvant immunotherapy combined with chemotherapy did not result in an increase in surgery-related risk and postoperative complications. Notably, the neoadjuvant group had significant objective tumor regression and pathological remission, thus, carrying a clinical value in guiding the designation of subsequent treatment regimens. Several studies have confirmed the good clinical and safety outcomes of neoadjuvant immunotherapy in combination with chemotherapy in patients with resectable ESCC [28], but the long-term survival benefit needs further investigation [29]. This study group plans to conduct a long-term follow-up investigation to verify the therapy’s ultimate efficacy [30, 31].

## Data Availability

The authors are accountable for all aspects of the work in ensuring that questions related to the accuracy or integrity of any part of the work are appropriately investigated and resolved. The datasets used are available from the corresponding author on reasonable request.

## Declarations

### Additional statements

All authors agree to participate and publish.
